# Comparison of Accessibility, Cost, and Quality of Elective Coronary Revascularization Between Veterans Affairs and Community Care Hospitals

**DOI:** 10.1001/jamacardio.2017.4843

**Published:** 2018-01-03

**Authors:** Paul G. Barnett, Juliette S. Hong, Evan Carey, Gary K. Grunwald, Karen Joynt Maddox, Thomas M. Maddox

**Affiliations:** 1 Veterans Affairs Health Economics Resource Center, VA Palo Alto Health Care System, Menlo Park, California; 2 Veterans Affairs Center for Innovation to Implementation, Menlo Park, California; 3 Center for Primary Care and Outcomes Research, Stanford University, Stanford, California; 4 Department of Biostatistics and Informatics, Colorado School of Public Health, University of Colorado, Anschutz Medical Campus, Aurora; 5 Veterans Affairs Eastern Colorado Health Care System, Denver; 6 Cardiology Division, John T. Milliken Department of Internal Medicine, Washington University School of Medicine in St Louis, St Louis, Missouri

## Abstract

**Importance:**

The Veterans Affairs (VA) Community Care (CC) Program supplements VA care with community-based medical services. However, access gains and value provided by CC have not been well described.

**Objectives:**

To compare the access, cost, and quality of elective coronary revascularization procedures between VA and CC hospitals and to evaluate if procedural volume or publicly reported quality data can be used to identify high-value care.

**Design, Setting, and Participants:**

Observational cohort study of veterans younger than 65 years undergoing an elective coronary revascularization, controlling for differences in risk factors using propensity adjustment. The setting was VA and CC hospitals. Participants were veterans undergoing elective percutaneous coronary intervention (PCI) and veterans undergoing coronary artery bypass graft (CABG) procedures between October 1, 2008, and September 30, 2011. The analysis was conducted between July 2014 and July 2017.

**Exposures:**

Receipt of an elective coronary revascularization at a VA vs CC facility.

**Main Outcomes and Measures:**

Access to care as measured by travel distance, 30-day mortality, and costs.

**Results:**

In the 3 years ending on September 30, 2011, a total of 13 237 elective PCIs (79.1% at the VA) and 5818 elective CABG procedures (83.6% at the VA) were performed in VA or CC hospitals among veterans meeting study inclusion criteria. On average, use of CC was associated with reduced net travel by 53.6 miles for PCI and by 73.3 miles for CABG surgery compared with VA-only care. Adjusted 30-day mortality after PCI was higher in CC compared with VA (1.54% for CC vs 0.65% for VA, *P* < .001) but was similar after CABG surgery (1.33% for CC vs 1.51% for VA, *P* = .74). There were no differences in adjusted 30-day readmission rates for PCI (7.04% for CC vs 7.73% for VA, *P* = .66) or CABG surgery (8.13% for CC vs 7.00% for VA, *P* = .28). The mean adjusted PCI cost was higher in CC ($22 025 for CC vs $15 683 for VA, *P* < .001). The mean adjusted CABG cost was lower in CC ($55 526 for CC vs $63 144 for VA, *P* < .01). Neither procedural volume nor publicly reported mortality data identified hospitals that provided higher-value care with the exception that CABG mortality was lower in small-volume CC hospitals.

**Conclusions and Relevance:**

In this veteran cohort, PCIs performed in CC hospitals were associated with shorter travel distance but with higher mortality, higher costs, and minimal travel savings compared with VA hospitals. The CABG procedures performed in CC hospitals were associated with shorter travel distance, similar mortality, and lower costs. As the VA considers expansion of the CC program, ongoing assessments of value and access gains are essential to optimize veteran outcomes and VA spending.

## Key Points

**Question:**

Does the Veterans Affairs Community Care Program, which allows veterans to receive care at non–Veterans Affairs sites, increase the accessibility and value of their elective coronary revascularization procedures?

**Findings:**

Among 13 237 elective percutaneous coronary interventions and 5818 elective coronary artery bypass graft procedures in this veteran cohort study, use of the Community Care Program reduced aggregate veteran travel distance for revascularization. Community Care Program hospitals had higher mortality and costs for percutaneous coronary intervention and had equivalent mortality and lower costs for coronary artery bypass graft surgery.

**Meaning:**

In our veteran cohort, use of Community Care Program hospitals improved overall access for revascularization; Community Care Program hospitals provided lower-value percutaneous coronary intervention procedures but higher-value coronary artery bypass graft procedures.

## Teaser

This veteran cohort study compares the access, cost, and quality of elective coronary revascularization procedures between Veterans Affairs and Community Care Program hospitals and evaluates if procedural volume or publicly reported quality data can be used to predict high-value care.

## Introduction

The US Department of Veterans Affairs (VA) supplements its hospitals and clinics with care purchased from community providers. This initiative, known as the Community Care Program (CC), cost $5.6 billion in 2014, representing 10% of the VA health care budget.^1^ The Veterans’ Access to Care through Choice, Accountability, and Transparency Act of 2014 expanded this program with a 3-year appropriation of $10 billion, with further expansions proposed.^2^ Currently, veterans are eligible to use CC services if the VA cannot provide necessary services due to a lack of available specialists, long wait times, or extraordinary distance from a veteran’s home.^5^

Elective coronary revascularization procedures, including percutaneous coronary intervention (PCI) and coronary artery bypass graft (CABG) procedures, accounted for $170 million of CC costs in 2014 (Health Economics Resource Center, unpublished data, February 2016). 

Understanding the influence of the CC program on access, outcomes, and costs is critical to the future of US veterans’ health care. We addressed 4 questions. First, did CC improve veterans’ access to elective coronary revascularization procedures by reducing travel distance and cost? Second, was the quality of care at CC hospitals comparable to that at VA hospitals? Third, was the cost of care lower at CC hospitals than at VA hospitals? Fourth, could the value of care be improved by selecting hospitals using publicly available information on factors associated with the quality of care, including annual procedure volumes and publicly reported risk-adjusted mortality?

## Methods

### Study Cohort

In this veteran cohort study, we evaluated patients younger than 65 years who had an elective coronary revascularization (PCI or CABG) sponsored by the VA between October 1, 2008, and September 30, 2011. Analysis was conducted between July 2014 and July 2017. Patients 65 years or older were excluded because they frequently use Medicare benefits to obtain coronary revascularization.^6^ Nonelective coronary revascularizations (ie, those receiving the procedure for either an acute myocardial infarction [AMI] or unstable angina) were also excluded because the urgency of these conditions generally requires treatment at the closest facility. We selected the first qualifying procedure of each patient. When an index stay involved transfer between hospitals, the procedure was attributed to the first hospital. Study procedures, waiver of informed consent, and waiver of Health Insurance Portability and Accountability Act of 1996 authorization were approved by the VA Central Institutional Review Board.

### Outcomes

Outcomes of interest were evaluated. These included access to care (as measured by travel distance), 30-day all-cause mortality, 30-day readmission for a cardiac-related diagnosis, and cost.

Access was measured as the additional travel distance required by the VA care arrangements: the distance traveled to the hospital that provided the procedure minus the distance to the nearest hospital offering that procedure (either VA or CC). Actual road distance and travel time between patient residence and hospital were calculated using a software program (ArcGIS; Esri). Zip code centroid was used for 3.0% of the cohort members with an incomplete address. There was no residential address for 3.1% of the cohort, and they were excluded from the travel analysis. We estimated all travel costs as if they were fully reimbursed by the VA regardless of current VA reimbursement practices. Travel cost was estimated at 41.5 cents per mile,^7^ the time patient and caregiver spent in transit valued at the federal minimum wage,^8^ plus lodging cost for those who traveled at least 40 miles. This cost was one night of lodging for patients obtaining outpatient PCI or one night of caregiver lodging for each night of hospital stay, to a maximum of 60 days, at the federal reimbursement rate for the county where the hospital was located.^9^

Date of death was determined from the VA Vital Status File. Hospital readmissions were considered cardiac related if they were stays in acute medical-surgical units that were assigned a principal diagnosis for heart disease, a specific procedural complication, cerebrovascular disease, renal failure, embolism or thrombosis of extremities, or other conditions plausibly related to complications or failure of coronary revascularization (eTable in the Supplement).

Health care costs included both hospital and professional components for the index admission and cardiac-related readmissions within 30 days. Costs at VA hospitals were obtained from the VA Managerial Cost Accounting System, an activity-based costing repository.^10^ Activity-based costing combines activity reports, financial data, workload, and intermediate products used in encounters and hospitals stays^11^ and is regarded as a more accurate measure of resource use than cost-adjusted charges.^12^ Costs for CC were the actual amount that the VA paid CC providers. All costs were adjusted to 2011 US dollars using the urban Consumer Price Index for all items.^14^

### Patient Data

Procedures, demographics, and medical comorbidities were obtained from CC claims data and from the national repository of VA electronic medical records in the 24 months before the index procedure. Both sources were used for all cohort members regardless of where the index procedure took place.

Procedural risk factors prevalent among elective patients were selected from validated models published by national cardiac registries for PCI^15^ and CABG surgery.^16^ Risk factors included age, sex, race/ethnicity, recent myocardial infarction, prior PCI, prior CABG surgery, cerebrovascular disease, peripheral vascular disease, congestive heart failure, diabetes (both type 1 and type 2), body mass index, renal function, dialysis, chronic obstructive pulmonary disease, atrial fibrillation, and severity of ischemic heart disease as represented by the number of vessels revascularized according to procedure coding.

Race was categorized into white, African American, or other based on patient self-report using best practices for VA data.^17^ Type 1 diabetes was based on prescription data. Body mass index was calculated as weight in kilograms divided by height in meters squared. Renal function was based on the estimated glomerular filtration rate derived from plasma creatinine.^18^ Left ventricular ejection fraction was obtained via natural language processing of VA echocardiogram reports, nuclear cardiac study reports, and other text entries in the electronic medical record.^19^ More detailed information from the VA Cardiac Catheterization Registry was consistently available only for patients receiving a procedure at the VA and hence was not used.

### Hospital Data

We considered proxy measures of quality that the VA might use for selective contracting, including volume and publicly reported mortality. Annual volume of PCI at CC hospitals was obtained from the National Cardiovascular Data Registry^20^ and from the 2010 to 2012 surveys of The Leapfrog Group.^21^ Annual volume of CABG surgery at CC hospitals was imputed using The Leapfrog Group survey and public Medicare data, including discharges for CABG surgery, total discharges, and percentage of revenue from Medicare. Annual volumes of PCI and CABG surgery at VA hospitals were obtained by tabulation of VA administrative data. Low-volume hospitals were defined as those performing fewer than 200 procedures per year for PCI^22^ and fewer than 125 procedures per year for CABG surgery.^23^ Publicly reported mortality for AMI was from the Medicare Hospital Compare report^24^ for 2011. Hospitals were defined as having high mortality risk if their risk-adjusted 30-day mortality was among the highest 10% reported to Hospital Compare.

### Statistical Analysis

The primary analysis compared travel distance and cost, mortality, readmission, and cost of care for patients undergoing procedures at VA vs CC hospitals. Secondary analyses considered if these outcomes differed in hospitals distinguished by proxy measures of quality, including low annual procedure volumes and high risk-adjusted mortality after AMI.

Estimation was by generalized estimating equations to account for clustering of patients within facilities.^25^ Differences in mortality and readmission were estimated with log binomial models and expressed as relative risk (RR), a measure that is more easily interpreted than the odds ratio.^27^ Log gamma models were used to accommodate the skewed distribution and heteroscedastic errors of costs.^28^ Because patients were not randomly assigned to VA or CC hospitals, propensity weighting was used to control for differences in case mix between VA and CC patients. Models were weighted by the inverse of the probability of receiving the treatment that the individual actually received.^30^ Propensity weights were based on logistic regressions of propensity to use CC, with separate models for PCI and CABG surgery. Dependent variables included the risk factors listed in the Patient Data subsection above with the exception of ejection fraction (because 42.4% of PCI cases at CC were missing ejection fraction data). The distribution of propensity scores across exposures was checked for balance and overlap. Overlap in predicted probabilities suggested that propensity analysis was appropriate for PCI (range, 0.08-0.49 for CC patients and 0.07-0.48 for VA patients) and CABG surgery (range, 0.08-0.46 for CC patients and 0.07-0.44 for VA patients). Trimming was deemed unnecessary. Unadjusted absolute value of standardized differences between VA and CC exceeded 10% for 3 of 19 covariates for PCI and for 7 of 19 covariates for CABG surgery. After applying inverse probability weights, all standardized differences had an absolute value of less than 10%, a frequently used criterion for adequacy of covariate balancing.^31^

## Results

### Study Cohort

Between October 1, 2008, and September 30, 2011, a total of 13 237 elective PCIs were performed in either VA or CC hospitals among veterans meeting study inclusion criteria (eFigure 1 in the Supplement). During the same period, 5818 patients underwent elective CABG procedures and met inclusion criteria (eFigure 2 in the Supplement).

### Characteristics of Patients and Hospitals

Veterans Affairs hospitals provided 10 474 (79.1%) of all PCIs in the study (Table 1). The VA and CC patients had a similar case mix, with some exceptions. Compared with CC patients, VA patients undergoing PCI were more likely to have congestive heart failure (21.1% vs 18.8%, *P* = .01) and more likely to have multivessel procedures. The CC patients were more likely to have renal impairment (estimated glomerular filtration rate <15 mL/min/1.73∙m^2^ or receiving dialysis in 4.5% vs 2.1%, *P* < .001) and an ejection fraction less than 30% (7.5% vs 5.7%, *P* = .005).

**Table 1.** Characteristics of Patients, Procedures, and Hospitals

Variable	PCI			CABG		
	VA (n = 10 474)	CC (n = 2763)	*P* Value^a^	VA (n = 4866)	CC (n = 952)	*P* Value^a^
**Demographics**						
Age, mean (SD), y	59.3 (5.0)	59.1 (5.4)	.13	59.8 (4.5)	59.7 (4.6)	.23
Age range, %						
<55 y	17.3	17.4	.97	13.7	14.5	.53
55 to <60 y	25.1	27.0	.04	24.7	24.8	.94
60 to <65 y	57.6	55.7	.07	61.6	60.7	.61
Male, %	97.9	98.0	.70	98.8	98.8	.92
Hispanic ethnicity, %	3.4	3.1	.43	5.0	3.1	.01
Race, %						
White	83.3	82.5	.37	84.5	85.7	.33
African American	14.1	14.6	.55	12.7	10.7	.09
Other	2.6	2.9	.42	2.8	3.6	.21
**Clinical History**						
Recent myocardial infarction, %	18.6	17.6	.22	20.5	22.2	.24
Prior PCI, %	20.5	19.9	.55	9.1	14.0	<.001
Prior CABG surgery, %	4.6	5.4	.08	0.3	0.6	.13
Cerebrovascular disease, %	14.9	15.5	.45	23.9	18.0	<.001
Peripheral vascular disease, %	22.5	22.4	.88	21.9	20.8	.45
Congestive heart failure, %	21.1	18.8	.01	23.1	24.9	.24
Ejection fraction, mean (SD)	52.5 (12.4)	51.7 (13.0)	.17	51.2 (12.0)	49.3 (13.6)	.004
Ejection fraction range, %						
≥52%	61.4	61.7	.85	56.9	50.4	<.001
41% to <52%	21.5	18.5	.008	23.7	24.5	.64
30% to <41%	11.5	12.3	.33	13.3	14.3	.46
<30%	5.7	7.5	.005	6.1	10.8	<.001
Diabetes, %	46.8	48.2	.17	49.2	47.5	.32
Type 2	31.6	31.8	.77	35.3	30.7	.006
Type 1	15.2	16.4	.12	14.0	16.8	.02
BMI, mean (SD)	31.2 (6.1)	30.9 (6.4)	.03	30.5 (5.7)	30.4 (6.0)	.28
BMI range, %						
<18	0.4	0.7	.008	0.3	0.3	.87
18 to <25	13.4	15.6	.004	14.5	17.3	.02
25 to <30	32.9	32.3	.59	35.7	34.5	.48
30 to <40	45.2	42.6	.01	43.2	41.6	.36
≥40	8.2	8.8	.29	6.3	6.3	.99
eGFR, mean (SD), mL∙min∙1.73∙m^2^	78.6 (21.1)	76.6 (24.7)	.01	78.5 (21.8)	76.8 (21.0)	.009
eGFR range, mL∙min∙1.73∙m^2^, %						
≥90	35.3	34.9	.67	36.8	31.8	.004
60 to <90	47.5	45.2	.03	45.9	48.8	.10
45 to <60	10.9	10.1	.23	10.3	12.0	.12
30 to <45	3.3	4.0	.10	3.8	4.7	.18
15 to <30	1.1	1.8	.002	1.3	1.3	.99
<15 or Dialysis	2.1	4.5	<.001	2.1	1.7	.41
Chronic obstructive pulmonary disease, %	28.7	30.1	.14	29.7	34.9	.001
Atrial fibrillation, %	7.0	8.2	.03	13.0	8.1	<.001
No. of vessels revascularized, %						
1	80.8	91.0	<.001	30.4	28.5	.23
2	15.0	6.6	<.001	41.8	33.6	<.001
3	3.2	2.0	<.001	22.2	27.0	.001
≥4	1.0	0.5	.02	5.6	10.9	<.001
Outpatient PCI, %	47.7	54.1	<.001	NA	NA	NA
**Hospital Characteristics**						
Annual PCI volume, mean (SD)	270.7 (140.5)	891.0 (728.3)	<.001	NA	NA	NA
Annual PCI volume <200, %	41.7	3.7	<.001	NA	NA	NA
Annual CABG volume, mean (SD)	NA	NA	NA	121.8 (48.9)	249.2 (166.7)	<.001
Annual CABG volume <125, %	NA	NA	NA	64.4	27.4	<.001
High AMI mortality risk, %	9.9	14.0	<.001	10.0	10.1	.91

Abbreviations: AMI, acute myocardial infarction; BMI, body mass index (calculated as weight in kilograms divided by height in meters squared); CABG, coronary artery bypass graft; CC, Community Care Program; eGFR, estimated glomerular filtration rate; NA, not applicable; PCI, percutaneous coronary intervention; VA, Veterans Affairs.

^a^ Wilcoxon rank sum test for continuous variables and χ^2^ test for categorical variables.

The VA patients were much more likely to receive PCI from a hospital that did not meet the recommended volume threshold of 200 cases annually (41.7% for VA vs 3.7% for CC, *P* < .001) (Table 1). In contrast, fewer VA patients received PCI in a hospital that had high risk-adjusted AMI mortality according to Hospital Compare (9.9% for VA vs 14.0% for CC, *P* < .001).

The VA hospitals provided 4866 (83.6%) of all CABG procedures in the study cohort. Compared with CC patients, VA patients undergoing CABG surgery were more likely to have atrial fibrillation (13.0% vs 8.1%, *P* < .001) (Table 1). The CC patients were more likely to have undergone prior PCI (14.0% vs 9.1%, *P* < .001), type 1 diabetes (16.8% vs 14.0%, *P* = .02), 3-vessel and 4-vessel procedures, or an ejection fraction less than 30% (10.8% vs 6.1%, *P* < .001).

The VA patients were more likely to receive CABG surgery at a hospital that did not meet the recommended volume threshold of 125 cases annually (64.4% for VA vs 27.4% for CC, *P* < .001) (Table 1). There was no difference between VA and CC in the proportion of patients who received CABG surgery in a hospital with high risk-adjusted AMI mortality (10.0% for VA vs 10.1% for CC, *P* = .91).

### Access

Relative to the nearest hospital offering PCI, the adjusted mean extra travel distance was 18.6 miles for CC patients and 72.2 miles for VA patients (*P* < .001) (Table 2). The mean adjusted cost of this additional travel, including the value of patient and caregiver time and lodging expense, was $34 for CC patients and $187 for VA patients (*P* < .001). Patients who used CC for PCI traveled a net distance that was, on average, 53.6 miles less and incurred a mean of $153 less in travel expense.

**Table 2.** Adjusted Travel Distance and Patient and Caregiver Travel Cost

Variable	PCI			CABG		
	VA	CC	*P* Value	VA	CC	*P* Value
**Travel Distance, miles**						
Actual distance traveled	90.8	60.1	<.001	123.2	81.5	.02
Distance to the nearest hospital	18.5	41.5	<.001	22.2	53.8	<.001
Extra travel distance	72.2	18.6	<.001	101.0	27.7	<.001
**Travel Cost, 2011 US $**						
Actual travel cost incurred	238	198	.004	958	630	<.001
Cost of travel to the nearest hospital	50	167	<.001	210	574	<.001
Extra travel cost incurred	187	34	<.001	747	57	<.001

Abbreviations: CABG, coronary artery bypass graft; CC, Community Care Program; PCI, percutaneous coronary intervention; VA, Veterans Affairs.

Relative to the nearest hospital offering CABG surgery, the adjusted mean extra travel distance was 27.7 miles for CC patients and 101.0 miles for VA patients (*P* < .001) (Table 2). The cost of this additional travel was $57 for CC patients and $747 for VA patients (*P* < .001). Patients who used CC for CABG surgery traveled a net distance that was an average of 73.3 miles less and incurred an average of $690 less in travel expense.

### Mortality and Readmissions

**Figure 1.**
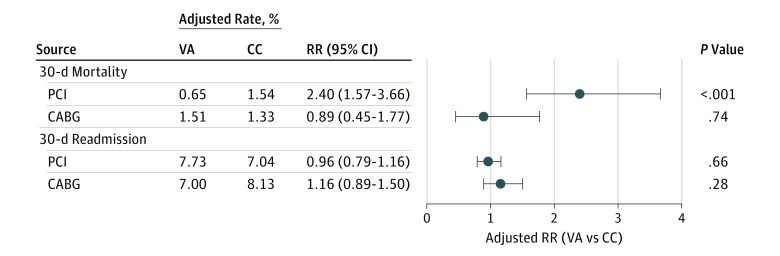
Adjusted 30-Day Mortality and Readmission Rates in Veterans Affairs (VA) and Community Care Program (CC) Hospitals The adjusted risk of 30-day mortality for elective percutaneous coronary intervention (PCI) was significantly elevated for CC hospitals compared with VA hospitals. There were no differences in adjusted 30-day mortality risk after elective coronary artery bypass graft (CABG) surgery or in risk of 30-day readmission. Covariates used for propensity adjustment included age, sex, race/ethnicity, recent myocardial infarction, prior PCI, prior CABG surgery, cerebrovascular disease, peripheral vascular disease, congestive heart failure, type 1 and type 2 diabetes, body mass index, renal function, dialysis, chronic obstructive pulmonary disease, atrial fibrillation, and the number of vessels revascularized. RR indicates relative risk.

Unadjusted 30-day mortality after PCI was 1.63% in CC hospitals and 0.63% in VA hospitals (45 deaths for CC vs 66 deaths for VA, *P* < .001). After propensity adjustment, 30-day mortality was higher for patients treated in CC hospitals compared with VA hospitals (1.54% vs 0.65%; RR, 2.40; 95% CI, 1.57-3.66) (Figure 1).

Unadjusted 30-day mortality after CABG surgery was 1.26% in patients treated in CC hospitals and 1.50% in patients treated in VA hospitals (12 deaths for CC vs 77 deaths for VA, *P* = .57). After propensity adjustment, 30-day mortality was similar for patients treated in CC hospitals compared with VA hospitals (1.33% vs 1.51%; RR, 0.89; 95% CI, 0.45-1.77) (Figure 1).

Unadjusted 30-day readmission rate after PCI was 7.14% for CC and 7.78% for VA (215 readmissions for CC and 748 readmissions for VA). Propensity-adjusted RR was 0.96 (95% CI, 0.79-1.16; *P* = .66) (Figure 1). Unadjusted readmission rate after CABG surgery was 8.25% for CC and 7.12% for VA (79 readmissions for CC and 346 readmissions for VA). Propensity-adjusted RR was 1.16 (95% CI, 0.89-1.50; *P* = .28).

### Cost

The mean adjusted cost of the index PCI procedure was $22 025 in CC hospitals and $15 683 in VA hospitals (*P* < .001) (Table 3). Total costs for patients undergoing PCI (the sum of the index procedure, readmission, and travel cost) were also significantly higher in CC hospitals ($23 059 vs $16 771, *P* < .001). The mean adjusted cost of the index CABG procedures was $55 526 in CC hospitals and $63 144 in VA hospitals (*P* < .01). Total costs were also significantly lower in CC hospitals ($56 749 vs $65 264, *P* < .01).

**Table 3.** Adjusted Costs by Procedure, Type of Cost, and Hospital Type

Variable	PCI, Mean (SD), 2011 US $			CABG, Mean (SD), 2011 US $		
	VA	CC	*P* Value	VA	CC	*P* Value
Cost of the index procedure	15 683 (16 493)	22 025 (30 701)	<.001	63 144 (46 018)	55 526 (74 797)	<.01
Cost of readmission	934 (4883)	968 (10 149)	.76	1215 (8682)	990 (9904)	.44
Extra travel cost	187 (238)	34 (324)	<.001	747 (762)	57 (699)	<.001
Total cost	16 771 (17 616)	23 059 (32 822)	<.001	65 264 (47 978)	56 749 (77 283)	<.01
	**Standard Volume**	**Low Volume**		**Standard Volume**	**Low Volume**	
Cost of the index procedure	19 697 (22 562)	16 006 (15 654)	<.001	59 261 (53 298)	59 419 (50 997)	.57
Cost of readmission	981 (6939)	847 (5008)	.27	1195 (10 602)	987 (7436)	.45
Extra travel cost	98 (281)	152 (243)	<.01	303 (805)	517 (832)	.09
Total cost	20 777 (24 017)	17 015 (16 883)	<.001	60 930 (56 044)	60 999 (52 724)	.60
	**Standard AMI Mortality**	**High AMI Mortality**		**Standard AMI Mortality**	**High AMI Mortality**	
Cost of the index procedure	18 373 (19 300)	22 430 (28 613)	.09	60 184 (53 556)	51 760 (33 520)	.13
Cost of readmission	987 (6571)	682 (4110)	.03	1128 (9280)	875 (3968)	.41
Extra travel cost	115 (276)	81 (209)	.96	407 (842)	325 (678)	.34
Total cost	19 453 (20 843)	23 364 (29 130)	.10	61 891 (55 840)	52 767 (34 146)	.11

Abbreviations: AMI, acute myocardial infarction; CABG, coronary artery bypass graft; CC, Community Care Program; PCI, percutaneous coronary intervention; VA, Veterans Affairs.

### Selection of Hospitals Using Proxy Measures

**Figure 2.**
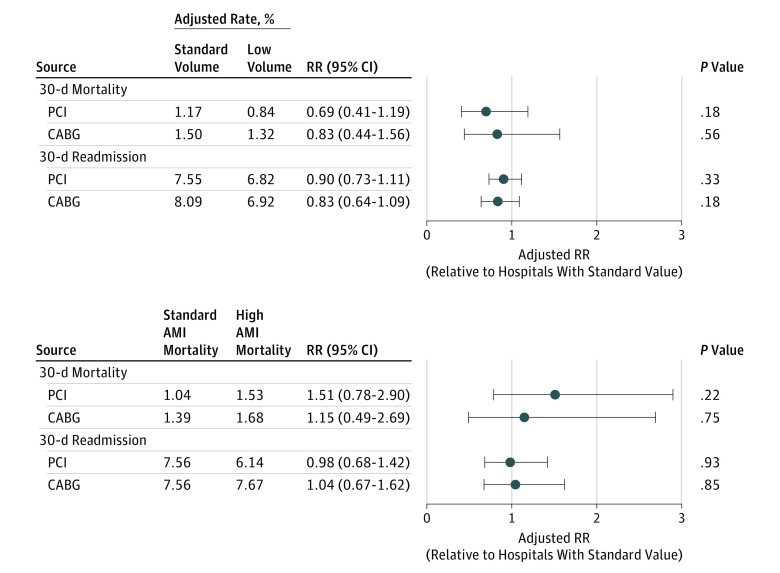
Adjusted 30-Day Mortality and Readmission Rates by Proxy Measures of Hospital Quality Adjusted risk of 30-day mortality or 30-day admission was not elevated at hospitals with a proxy indicator of quality limitation, including annual procedure volume below the recommended standard or acute myocardial infarction (AMI) mortality risk in the upper 10% reported to Hospital Compare. CABG indicates coronary artery bypass graft; PCI, percutaneous coronary intervention; and RR, relative risk.

For either procedure, low-volume hospitals had similar mortality and readmission rates compared with hospitals that met the recommended volume standards (Figure 2). The cost of the index PCI procedure was significantly less at low-volume hospitals compared with standard-volume hospitals ($16 006 vs $19 697, *P* < .001) (Table 3). Low-volume hospitals were more likely to be operated by the VA and to provide a single-vessel procedure, which were factors associated with lower cost. Although travel cost was greater at low-volume hospitals, total costs of PCI were also significantly less at low-volume facilities. There were no significant differences in CABG cost.

Hospitals that reported high AMI mortality to Hospital Compare were compared with hospitals that reported standard mortality. For both procedures, mortality and readmission rates did not differ between these groups of hospitals. The cost of readmission after PCI was less at hospitals with high AMI mortality than at hospitals with standard AMI mortality ($682 vs $987, *P* = .03) (Table 3). There were no significant differences in CABG cost.

Selecting hospitals using proxy measures did not improve outcomes or reduce cost. Because there was no advantage to this strategy, we did not quantify how it would have increased the burden of patient travel.

Additional analyses tested if outcomes differed by the interaction between proxy measure and the type of hospital (VA or CC). The interaction was significant for CABG mortality for low-volume hospitals. Low-volume CC hospitals had significantly lower mortality after CABG surgery relative to standard-volume CC hospitals (RR, 0.15; 95% CI, 0.05-0.35), low-volume VA hospitals (RR, 0.15; 95% CI, 0.06-0.30), and standard-volume VA hospitals (RR, 0.21; 95% CI, 0.09-0.41). The only other interaction that was significant was the cost of the index PCI care for low-volume hospitals. The mean adjusted cost of the index PCI was $22 257 at standard-volume CC hospitals, $15 702 at low-volume CC hospitals, $16 031 at low-volume VA hospitals, and $15 431 at standard-volume VA hospitals (contrasts between CC high-volume hospitals and each of the others were significant at *P* < .001).

## Discussion

We studied elective procedures sponsored by the VA between 2008 and 2011 in patients younger than 65 years and found that 20.9% of PCIs and 16.4% of CABG procedures were performed at CC sites. The CC program was associated with significantly lower travel costs, with an average of $153 less travel cost for PCI and $690 less travel cost for CABG surgery. The value of this reduced travel must be balanced against differences in the quality of services and their cost. Herein, our findings were mixed. For PCI, VA hospitals had lower mortality (0.65% vs 1.54% for CC), similar readmission rates, and lower costs ($15 683 vs $22 025 for CC). For CABG surgery, VA hospitals had similar mortality, similar readmission rates, and higher cost ($63 144 vs $55 526 for CC).

The higher mortality of CC-provided PCIs was not necessarily due to lower quality of care at CC hospitals. Other possible factors include delay in making care arrangements, incomplete coordination of care between VA and CC hospitals, or failure to refill medications prescribed by CC clinicians. These are obvious areas for future research and quality improvement efforts. New VA data on scheduling of care, including a database of CC approvals, will help the VA detect problems associated with treatment delay.

We found that publicly available data on hospital volume and Medicare mortality did not reliably identify centers where veterans had better outcomes. The VA currently requires CC providers to have an active license and a lack of sanctions but does not set minimum quality thresholds or choose hospitals based on cost.^32^ Better information on the characteristics of CC patients and the hospitals that care for them could improve VA decision making. For this reason, we recommend that the VA seek information needed to assess the quality of care, including performance measures based on submission to the national registries of PCI and CABG surgery. This process could allow the VA to selectively contract with hospitals that meet standards of both quality and transparency.

Our findings also demonstrate that, on average, veterans seeking high-quality care, with low mortality and readmission rates, are well served by the VA. This outcome confirms the findings of prior studies finding that the quality of VA care is generally similar to that of non-VA hospitals,^6^ with some exceptions, particularly from older studies.^38^ Although many VA sites operate with annual procedure volumes that are less than the recommended minimum, VA mortality rates were low. This finding may be due to the influence of national VA programs to monitor outcomes and improve the quality of VA surgery^44^ and PCI.^37^ Therefore, one important way to improve value for veterans may be to increase capacity at high-performing VA facilities rather than seek to increase capacity by outsourcing to the community.

### Limitations

This study has several limitations. Mortality in our sample was a sufficiently rare event that the power to detect differences in the quality of care of VA and CC may have been limited. We only examined patients younger than 65 years, and our findings may not generalize to an older population. Our data only include procedures between 2008 and 2011, and patterns may have changed over time. We did not study the influence of waiting time on outcomes because we did not have information on when procedures were first recommended. We attempted to address this issue by limiting the study to elective procedures, which are not as time sensitive as emergent procedures.

While VA and CC patients were similar in terms of coded comorbidities, it is possible that there was undetected referral bias such that patients referred to CC hospitals had elevated risk beyond that represented by the available covariates. Our comparison was limited by the lack of information on CC patients. We had information on the VA registry of patients undergoing PCI, but data for CC patients were not available because of the restrictions governing the national registry of patients undergoing PCI.

Finally, we did not have the clinical detail needed to ascertain if procedures were appropriate. Such an evaluation will be needed for a complete assessment of the value of CC.

## Conclusions

In summary, our study found that almost 1 in 5 elective coronary revascularizations for VA patients was performed at CC sites. The VA hospitals had lower mortality and lower costs than CC hospitals for PCI and had similar mortality but higher costs for CABG surgery. To ensure that veterans receive care that is timely, accessible, and of the highest quality, policymakers should consider providing information to help veterans seek care from the highest-value hospitals and health care professionals regardless of whether the hospitals are VA or CC.

## Notes

Supplement. **eTable.** Principal Diagnoses Used to Identify Readmissions That Were Cardiac Related **eFigure 1.** PCI Cohort Definition **eFigure 2.** CABG Cohort Definition
