# Supplementary material for: Comparison of Accessibility, Cost, and Quality of Elective Coronary Revascularization Between Veterans Affairs and Community Care Hospitals
Source: JAMA Cardiol. 2018 Jan 3;3(2):133–41. doi: 10.1001/jamacardio.2017.4843 (PMC5838592; doi:10.1001/jamacardio.2017.4843)
Supplement: Supplement. — eTable. Principal Diagnoses Used to Identify Readmissions That Were Cardiac Related eFigure 1. PCI Cohort Definition eFigure 2. CABG Cohort Definition [file jamacardiol-3-133-s001.pdf]

Supplementary Online Content

Barnett PG, Hong JS, Carey E, Grunwald GK, Joynt Maddox K, Maddox TM. Comparison of accessibility, cost, and quality of elective coronary revascularization between Veterans Affairs and Community Care Hospitals. *JAMA Cardiol*. Published online January 3, 2018. doi:10.1001/jamacardio.2017.4843

eTable. Principal Diagnoses Used to Identify Readmissions That Were Cardiac Related

eFigure 1. PCI Cohort Definition

eFigure 2. CABG Cohort Definition

This supplementary material has been provided by the authors to give readers additional information about their work.

© 2017 Barnett PG, et al. *JAMA Cardiology*.

eTable. Principal Diagnoses Used to Identify Readmissions That Were Cardiac Related

ICD-9 Code	Full Text Description
36845	Generalized visual field contraction or constriction
3689	Unspecified visual disturbance
3699	Unspecified visual loss
37741	Ischemic optic neuropathy
4010	Malignant essential hypertension
4019	Unspecified essential hypertension
40201	Hypertensive heart disease, malignant, with heart failure
40390	Hypertensive chronic kidney disease, unspecified, with chronic kidney disease stage i through stage iv, or unspecified
40391	Hypertensive chronic kidney disease, unspecified, with chronic kidney disease stage v or end stage renal disease
40491	Hypertensive heart and chronic kidney disease, unspecified, with heart failure and with chronic kidney disease stage i through stage iv, or unspecified
41001	Acute myocardial infarction, of anterolateral wall, initial episode of care
41002	Acute myocradial infarction, of anterolateral wall, subsequent episode of care
41011	Acute myocardial infarction, of other anterior wall, initial episode of care
41012	Acute myocardial infarction, of other anterior wall, subsequent episode of care
41021	Acute myocardial infarction, of inferolateral wall, initial episode of care
41022	Acute myocardial infarction, of inferolataeral wall, subsequent episode of care
41031	Acute myocardial infarction, of inferoposterior wall, initial episode of care
41041	Acute myocardial infarction, of other inferior wall, initial episode of care
41042	Acute myocardial infarction, of other inferior wall, subsequent episode of care
41051	Acute myocardial infarction, of other lateral wall, initial episode of care
41061	Acute myocardial infarction, true posterior wall infarction, initial episode of care
41070	Acute myocardial infarction, subendocardial infarction, episode of care unspecified
41071	Acute myocardial infarction, subendocardial infarction, initial episode of care
41072	Acute myocardial infarction, subendocardial infarction, subsequent episode of care
41080	Acute myocardial infarction, of other specified sites, episode of care unspecified
41081	Acute myocardial infarction, of other specified sites, initial episode of care
41090	Acute myocardial infarction, unspecified site, episode of care unspecified
41091	Acute myocardial infarction, unspecified site, initial episode of care
41092	Acute myocardial infarction, unspecified site, subsequent episode of care
4110	Postmyocardial infarction syndrome
4111	Intermediate coronary syndrome
41181	Acute coronary occlusion without myocardial infarction
4131	Prinzmetal angina
4139	Other and unspecified angina pectoris

© 2017 Barnett PG, et al. *JAMA Cardiology.*

ICD-9 Code	Full Text Description
41400	Coronary atherosclerosis of unspecified type of vessel, native or graft
41401	Coronary atherosclerosis of native coronary vessel
41402	Coronary atherosclerosis of autologous vein bypass graft
41403	Coronary atherosclerosis of nonautologous biological bypass graft
41404	Coronary atherosclerosis of artery bypass graft
41405	Coronary atherosclerosis of unspecified type of bypass graft
41406	Coronary atherosclerosis, of native coronary artery of transplanted heart
41412	Dissection of coronary artery
4148	Other specified forms of chronic ischemic heart disease
4149	Chronic ischemic heart disease, unspecified
42090	Acute pericarditis, unspecified
42099	Other acute pericarditis
4210	Acute and subacute bacterial endocarditis
4230	Hemopericardium
4233	Cardiac tamponade
4239	Unspecified disease of pericardium
42490	Endocarditis, valve unspecified, unspecified cause
4260	Atrioventricular block, complete
42613	Other second degree atrioventricular block
4270	Paroxysmal supraventricular tachycardia
4271	Paroxysmal ventricular tachycardia
4272	Paroxysmal tachycardia, unspecified
42731	Atrial fibrillation
42732	Atrial flutter
42741	Ventricular fibrillation
4275	Cardiac arrest
42769	Other premature beats
42781	Sinoatrial node dysfunction
42789	Other specified cardiac dysrhythmias
4279	Cardiac dysrhythmia, unspecified
4280	Congestive heart failure, unspecified
42820	Unspecified systolic heart failure
42821	Acute systolic heart failure
42822	Chronic systolic heart failure
42823	Acute on chronic systolic heart failure
42830	Unspecified diastolic heart failure
42831	Acute diastolic heart failure
42833	Acute on chronic diastolic heart failure.
42840	Unspecified combined systolic and diastolic heart failure

© 2017 Barnett PG, et al. *JAMA Cardiology*.

ICD-9 Code	Full Text Description
42841	Acute combined systolic and diastolic heart failure.
42843	Acute on chronic combined systolic and diastolic heart failure
4289	Heart failure, unspecified
430	Subarachnoid hemorrhage
431	Intracerebral hemorrhage
4321	Subdural hemorrhage
4329	Unspecified intracranial hemorrhage
43310	Occlusion & stenosis of carotid artery, w/o cerebral infarction
43311	Occlusion & stenosis of carotid artery, w/ cerebral infarction
43330	Occlusion & stenosis fo multiple & bilateral arteries, w/o mention of cerebral infarction
43331	Occlusion& stenosis of multiple & bilateral arteries, w/ cerebral infarction
43380	Occlusion & stenosis of other specified precerebral artery, w/o mention of cerebral infarction
43410	Cerebral embolism w/o mention of cerebral infarction
43411	Cerebral embolism w/ cerebral infarction
43490	Cerebral artery occlusion, unspecified, w/o mention of cerebral infarction
43491	Cerebral artery occlusion, unspecified, w/ cerebral infarction
4359	Unspecified transient cerebral ischemia
436	Acute, but ill-defined, cerebrovascular disease
4370	Cerebral atherosclerosis
4373	Cerebral aneurysm, nonruptured
43811	Late effect of cerebrovascular disease, speech and language deficits, aphasia
43820	Late effect of cerebrovascular disease, hemiplegia affecting uspecified side
43889	Other late effects of cerebrovascular disease
4401	Atherosclerosis of renal artery
44020	Atherosclerosis of native arteries of extremities, unspecified
44021	Atherosclerosis of native arteries of extremities w/intermittent claudication
44022	Atherosclerosis of native arteries of extremities with rest pain
44023	Atherosclerosis of native arteries of extremities w/ ulceration
44024	Atherosclerosis of native arteries of extremities w/ gangrene
44030	Atherosclerosis of unspecified bypass graft of the extremities
44031	Atherosclerosis of autologous vein bypass graft of the extremities
4408	Atherosclerosis of other specified arteries
44103	Dissection aorta, thoracoabdominal
4413	Abdominal aneurysm, ruptured
44322	Dissection of iliac artery
44421	Arterial embolism and thrombosis of upper extremity
44422	Arterial embolism and thrombosis of lower extremity
44481	Embolism and thrombosis of iliac artery
44489	Embolism and thrombosis of other artery

© 2017 Barnett PG, et al. *JAMA Cardiology*.

ICD-9 Code	Full Text Description
44502	Atheroembolism lower extremity
4580	Orthostatic hypotension
4581	Chronic hypotension
45829	Other iatrogenic hypotension
4589	Hypotension, unspecified
45989	Other specified circulatory system disorders
4599	Unspecified circulatory system disorder
53100	Acute gastric ulcer with hemorrhage, without mention of obstruction
53140	Chronic or unspecified gastric ulcer with hemorrhage, without mention of obstruction
53160	Chronic or unspecified gastric ulcer with hemorrhage and perforation, without mention of obstruction
53240	Chronic or unspecified duodenal ulcer with hemorrhage, without mention of obstruction
53340	Chronic or unspecified peptic ulcer of unspecified site with hemorrhage, without mention of obstruction
53541	Other specified gastritis w hemorrhage
53551	Unspecified gastritis and gastroduodenitis w hemorrhage
53561	Duodenitis with hemorrhage
53783	Angiodysplasia of stomacch and duodenum w hemorrhage
53784	Dielulafoy lesion (hemorrhagic) of stomach and duodenum
5570	Acute vascular insufficiency of intestine
5579	Unspecified vascular insufficiency of intestine
5780	Hematemesis
5781	Blood in stool
5789	Hemorrhage of gastrointestinal tract, unspecified
58089	Acute glomerulonephritis with other specified pathological lesion in kidney
5845	Acute renal failure with lesion of tubular necrosis
5849	Acute renal failure, unspecified
5854	Chronic kidney disease, stage iv (severe)
5856	End stage renal disease
586	Renal failure, unspecified
58889	Other specified disorders resulting from impaired renal function
59970	Hematuria, unspecified
59971	Gross hematuria
71916	Hemarthrosis involving lower leg
7802	Syncope and collapse
78551	Cardiogenic shock
99603	Mechanical complication due to coronary bypass graft
99661	Infection and inflammatory reaction due to cardiac device, implant, and graft
99672	Other complications due to other cardiac device, implant, and graft
99702	Iatrogenic cerebrovascular infarction or hemorrhage

© 2017 Barnett PG, et al. *JAMA Cardiology*.

ICD-9 Code	Full Text Description
9971	Cardiac complications, not elsewhere classified
9972	Peripheral vascular complications, not elsewhere classified
99779	Vascular complications of other vessels
99811	Hemorrhage complicating a procedure
9982	Accidental puncture or laceration during a procedure, not elsewhere classified
99883	Non-Healing surgical wound
99889	Other specified complications of procedures not elsewhere classified
V5873	Aftercare following surgery of the circulatory system, nec
V717	Observation for suspected cardiovascular disease

© 2017 Barnett PG, et al. *JAMA Cardiology*.

eFigure 1. PCI Cohort Definition. Of 59,925 procedures sponsored by VA between 2008 and 2011, 13,237 were included in the final cohort.

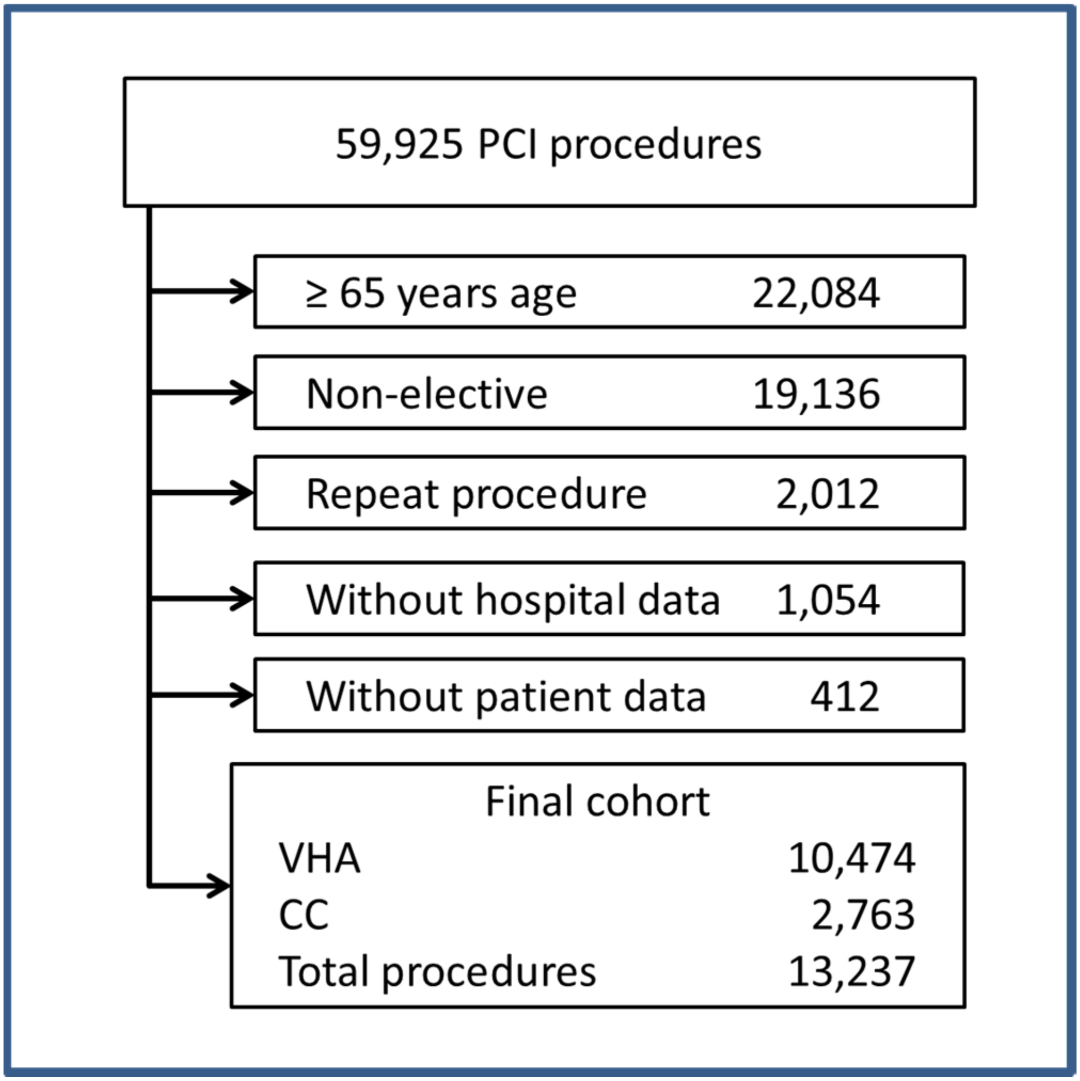

© 2017 Barnett PG, et al. *JAMA Cardiology*.

eFigure 2. CABG Cohort Definition. Of 18,816 CABG surgeries sponsored by VA between 2008 and 2011, 5,818 were included in the final cohort.

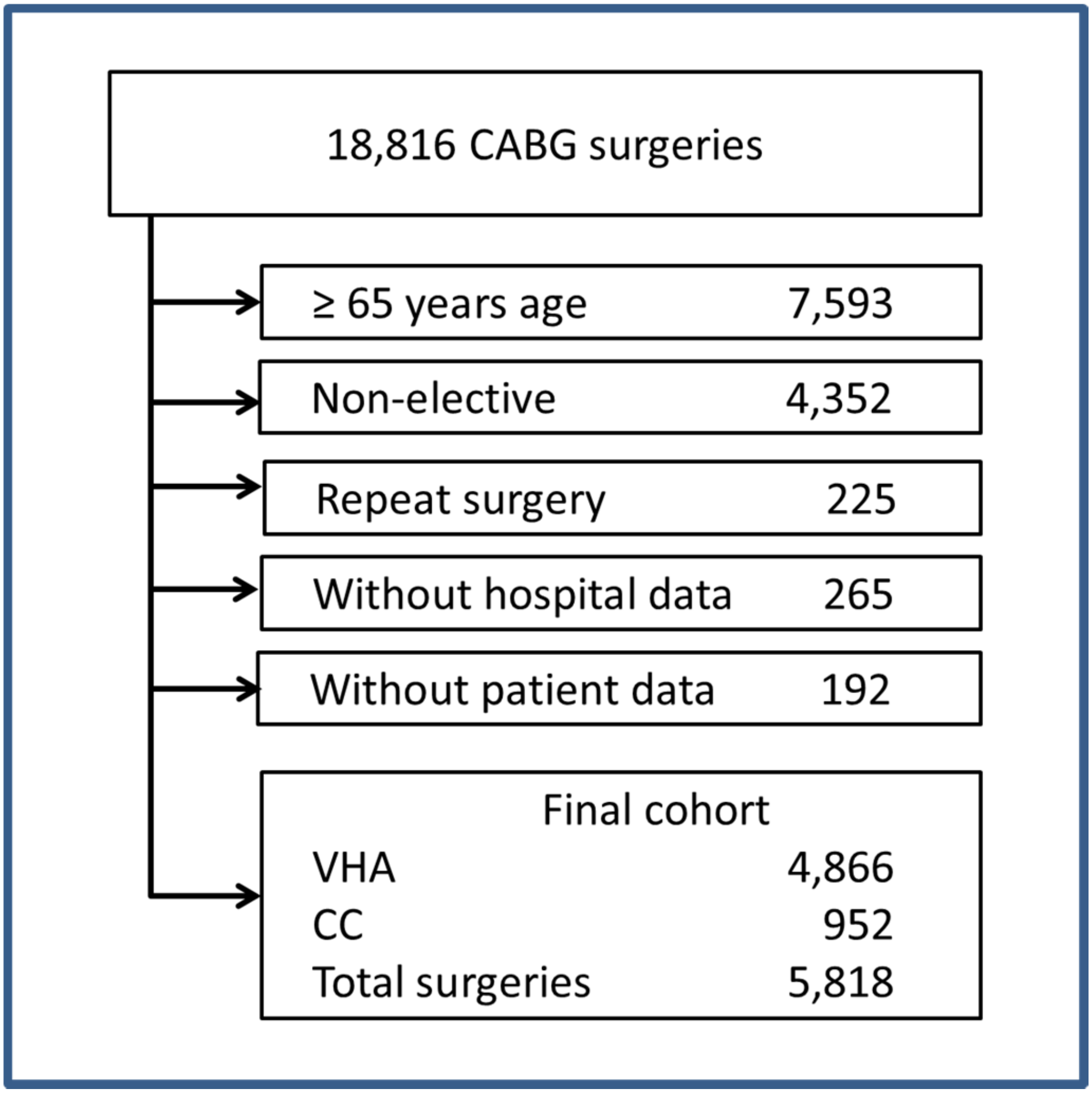

© 2017 Barnett PG, et al. *JAMA Cardiology*.
